# Infection of Hemothorax by Anaerobic Gas-producing Bacilli

**Published:** 1918-01

**Authors:** 


### SURGICAL

***Infection of Hemothorax by Anaerobic Gas-producing Bacilli.*** By T. R. Elliott, R. A. M. C. and Herbert Henry, R. A. M. C. Abs. from the *British Medical Journal*, March 31, 1917 and April 7, 1917.

The authors give a detailed account of their study of infected hemothorax. They find that infection by anaerobic bacilli occurs in about 10 o/o of all cases of hemothorax from wounds seen in base hospitals. Foul gas is abundantly formed giving rise sometimes to a large pneumohemothorax or to a loculted form. The toxic features of the infection may temporarily appear slight. The gas may be under considerable pressure. This induces and maintains complete collapse of the lung and may cause extreme displacement of the heart. Symptoms of cardiac and pulmonary embarrassment may be prominent. Septic features, however, are much more prominent, such as, jaundice, a rapid pulse (often an index of pericarditis) and semi-delirious restlessness. The febrile type, accompanied by a hectic flush on the cheeks, is less dangerous.

An exploratory puncture must first be made. In case of septic symptoms it is well to repeat the explorations on successive days, because the anaerobes often fail to spread through the whole hemothorax, and may easily be missed at first.

*Treatment.* — Drainage is the first essential. A day's delay in detecting the infection and establishing proper drainage may prove fatal. To help in diagnosis it should be remembered that hemothorax fluid never clots massively in the syringe or test tube, but that lung and liver blood do so. If this fluid is found sterile under the microscope, it is usually safe to wait and watch developments. " It is *never* justifiable to resort at once to rib resection and drainage merely because the patient presents all the clinical appearances of severe asepsis. " If there is no improvement, an exploration should be made on the following day, especially if on the first occasion the fluid has, upon centrifuging, yielded a rich field of polymorphonuclear cells. These are frequently a warning of the spread of the bacilli. If, however, there is an offensive odor from the sample withdrawn, surgical treatment is justified at once without waiting for further bacteriological investigation.

Treatment should aim: (1) to remove gas and infected fluid, (2) to remove the septic blood-clot, (3) to destroy the organisms,

(4) to remove any foreign bodies, (5) to provide for rapid reexpansion of the collapsed lung, and to prevent secondary infection.

It is as yet impossible to realize all of these objects. The operator must often content himself with the saving of life.

It has been observed that the organisms are very slow in developing in the hemothorax or in fresh blood or live tissues. If it were possible to treat a case by opening the wounded chest at once and removing the clot and all sources of infection, as is sometimes done, this line of treatment might be preferred. The conditions of war surgery, however, render such immediate treatment out of the question in most cases. The doctors usually find themselves confronted with an established infection.

The authors have generally tried to avoid opening the chest. They have treated the infection, when possible, by repeated aspirations combined with antiseptic lavage. Fortunately many of the organisms tend to die out in the body fluids. The authors have found injections of oxygen and closed lavage with eusol perfectly useless. They explain this failure on the ground that the clot has become practically a foreign body, and, if it has become septic, it must be removed.

When the blood clot has not become infected and the purulent reaction is slight, the body may overcome the organism if aided by the aspiration of the dead fluid. The authors have noted that the pleura has astonishing powers to dispose of infection. Therefore if aspirations are begun at an early date they may suffice.

In the vast majority of cases, however, the clot is infected, and drainage is absolutely necessary. The pleural cavity must be opened and the clots detached or washed away and any foreign body removed. So far it has not been found best to close the cavity at once. Drainage has to be maintained for accumulating suppuration.

Drainage should be contrived to empty the lowest recess of the acvity. In cases of large empyemata, without marked toxic features, it is well to employ airtight drainage, so as to avoid secondary infection, and to help in the reexpansion of the lung. The sewing of the parietal pleura, the enveloping muscle, and the skin around the tube requires about fifteen minutes. To create a negative pressure the free end of the tube may be dipped below the surface of a weak disinfectant, or an aspirating bottle may be used. The suction helps a little towards the expansion of the collapsed lung, and is at first a source of comfort to the patient. The respiratory movements of the patient, however, are far more important, and they should be deliberately practiced.

If open drainage is resorted to, the surgeon should aim to do away with the tube as early as possible. If, however, the clot has not been completely removed at first, and if offensive discharges continue, antiseptic lavage with eusol, carbolic acid, or hydrogen peroxide must be maintained in order to dislodge the clot. All accessible foreign bodies should be removed as soon as possible. The authors are not quite sure whether it is best to remove those in the substance of the lung at once, or to wait for them to " ulcerate their way to the surface ". They fear that this latter process may in some cases cause damage to a vessel and consequently produce secondary hemorrhage.

The authors believe that life may be saved in all cases except those with complicating wounds of the abdomen or with virulent and mixed infections. A dry tongue is a valuable indication of the latter. So long as the tongue remains moist the patient ought to recover. Death from sepsis almost never occurs after the end of the second week following the wound.

